# Achievement of Treatment Goals and Mortality in Individuals with Diabetes: The ELSA-Brasil Study

**DOI:** 10.3390/jcm12247663

**Published:** 2023-12-13

**Authors:** Bruna Cristine Chwal, Rodrigo Citton P. dos Reis, Maria Inês Schmidt, Sandhi Maria Barreto, Rosane Harter Griep, Bruce B. Duncan

**Affiliations:** 1 Postgraduate Program in Epidemiology, Universidade Federal do Rio Grande do Sul, R. Ramiro Barcelos, 2600/518, Porto Alegre CEP 90035-003, Brazil; brunacristine.chwal@gmail.com (B.C.C.); rodrigocpdosreis@gmail.com (R.C.P.d.R.); mischmidt49@gmail.com (M.I.S.); 2 Departamento de Estatística, Universidade Federal do Rio Grande do Sul, Porto Alegre CEP 90040-060, Brazil; 3 Hospital de Clínicas de Porto Alegre, Porto Alegre CEP 90035-903, Brazil; 4 Faculdade de Medicina e Hospital das Clínicas/EBSERH, Universidade Federal de Minas Gerais (UFMG), Belo Horizonte CEP 31270-901, Brazil; sandhi.barreto@gmail.com; 5 Laboratório de Educação em Ambiente e Saúde, Instituto Oswaldo Cruz, Fundação Oswaldo Cruz, Rio de Janeiro CEP 21040-360, Brazil; rohgriep@gmail.com

**Keywords:** diabetes mellitus, cardiometabolic risk factors, mortality, glycated hemoglobin A, hypertension, hypercholesterolemia, smoking

## Abstract

Background: To prevent diabetes complications, the American Diabetes Association (ADA) has recommended the treatment of blood glucose, blood pressure, and LDL-cholesterol (LDL-c) to target levels. Our aim is to characterize the risk of death according to the achievement of these goals in subjects with diabetes participating in the ELSA-Brasil study. Methods: ELSA-Brasil is an occupational cohort study of middle-aged and elderly adults followed from a 2008–2010 baseline to 2019 by two additional clinic visits and annual telephone interviews. We ascertained known diabetes by self-reported diagnosis or anti-diabetic medication use. We used treatment targets based on the 2022 ADA guidelines. We ascertained deaths from any cause based on the annual surveillance confirmed by death certificates. Results: After 11 (1.8) years of follow-up, 261 subjects had died among 2423 with known diabetes. Within-target HbA1c was associated with the greatest protection (HR = 0.66; 95%CI 0.50–0.88) against all-cause mortality. Achieving both glycemic and blood pressure targets conferred substantial protection (HR = 0.54; 95%CI 0.37–0.78). Within-target LDL-c, however, was associated with increased mortality (HR = 1.44; 95%CI 1.11–1.88). Conclusions: Glucose and blood pressure control, especially when concomitant, reduced mortality. The increased mortality associated with achieving the LDL-c target merits further investigation.

## 1. Introduction

Therapeutic strategies for those with diabetes targeting multiple factors can decrease the risk of complications, especially cardiovascular diseases [1]. A large Swedish study demonstrated the potential benefit gained from controlling hypertension, hyperglycemia, and hypercholesterolemia, along with albuminuria and smoking—those with diabetes having all of these factors in control approached the risk of death of those without the disease [2]. 

The treatment of diabetes has also changed significantly in recent years, as new medications with broad effects beyond just glycemic control have become available. Sodium-glucose cotransporter-2 (SGLT2) inhibitors and glucagon-like peptide-1 receptor agonists (GLP-1RA), as well as newer weight loss drugs, such as semaglutide and tirzepatide, are now recommended by authorities for many patients, given their proven benefit in terms of cardiovascular and renal outcomes and overall mortality [3,4].

In Brazil, where diabetes was recently ranked as the sixth leading cause of death [5], the attainment of the control of these factors is not satisfactory [6,7]. Further, in Brazil and other low- and middle-income countries (LMICs), the more recent and effective, though costly, medications for glucose control have yet to be widely available, emphasizing the continued necessity of evaluating the benefit of more traditional therapeutic options. The importance of this control with more conventional medications in LMICs is less well documented, as studies assessing mortality according to the achievement of treatment goals have been generally limited to small samples and hospital centers [8,9]. 

To contribute to closing this gap in the literature concerning the association of control using traditional medications with mortality in middle-income countries, we aimed to relate the achievement of blood glucose, hypertension, and LDL-cholesterol control with the risk of death in a large, contemporary cohort of Brazilian middle-aged and elderly adults with diabetes participating in the Brazilian Longitudinal Study of Adult Health (ELSA-Brasil).

## 2. Materials and Methods

### 2.1. Study Population and Ethics

The ELSA-Brasil cohort enrolled 15,105 current or retired civil servants aged 35 to 74 at public institutions of higher education and research located in the capital cities of Bahia, Espírito Santo, Minas Gerais, Rio de Janeiro, São Paulo, and Rio Grande do Sul [10]. We collected data in clinical research centers during three visits (2008–2010, 2012–2014, and 2017–2019) [10]. For this study, we included only participants with self-reported known diabetes or pharmacologic treatment for diabetes. Research ethics committees at each clinic center approved the study, and all participants gave written informed consent to participate.

### 2.2. Measurements 

Centrally trained and certified teams at ELSA research clinics conducted standardized interviews and clinical assessments and collected samples for biochemical tests at all visits [11]. 

The study obtained baseline data on age, sex, ethnicity (white, *pardo* [mixed], black, Asian, and indigenous), smoking, and history of a medical diagnosis of diabetes and anti-diabetic medication use through interviews. ELSA clinic staff performed three blood pressure measurements, and systolic and diastolic blood pressure were calculated as the mean of the last two [12,13]. Weight and height were obtained using a standardized protocol and body mass index was calculated as weight/height^2^ (kg/m^2^) [11]. Waist circumference and weight were measured while fasting and with an empty bladder. Participants wore standardized clothing without spectacles or other personal objects during measurement. Height was measured to the nearest 0.1 cm (Seca model SE-216, Hamburg, Germany). Waist circumference was assessed with a 150 cm inelastic measuring tape (Mabis-Gulick, Waukegan, IL, USA) placed in the mid-axillary line at the midpoint between the inferior edge of the costal border and the iliac crest. Body weight was measured with a balance-beam scale with a maximum capacity of 300 kg (Toledo, São Bernardo do Campo, Brazil) [11].

The study obtained blood samples after an overnight (>8 h) fast and corresponding overnight 12 h urine collection. The samples obtained were frozen and shipped to a central laboratory for determination. Plasma glucose was measured using the hexokinase method (Cobas c501^®^, Roche Diagnostics, Rotkreuz, Switzerland) and glycated hemoglobin (HbA1c) by high-pressure chromatography (HPLC—Bio-Rad Laboratories, Hercules, CA, USA). Cholesterol was determined by an enzymatic colorimetric method, and triglycerides were determined by glycerol-phosphate peroxidase (Cobas c501^®^, Roche Diagnostics). Low-density cholesterol was estimated (LDL-c) by the Friedewald equation when total triglycerides were <400 mg/dL (<4.51 mmol/L) and measured directly when triglycerides were ≥400 mg/dL (≥4.51 mmol/L). The kinetic Jaffe method (Advia 1200 Siemens, Tarrytown, NY, USA) was employed to measure creatinine, and the immunochemical assay (BN II Nephelometer Siemens Dade Behring, USA) was used to measure urine albumin. The urine albumin creatinine ratio was calculated from albumin and creatinine concentrations in 12 h overnight urine samples. Interclass correlations for glucose, HbA1c, LDL-c, creatinine, and albumin were 0.99, 0.94, 0.99, 0.93 and 1.00, respectively [14].

ELSA used the leisure domain of the validated Portuguese version of the International Physical Activity Questionnaire (IPAQ) long form to quantify physical activity. Physical activity was assessed in MET minutes/week by multiplying the weekly frequency of activities of a given intensity (walking, moderate, intense) by their duration and by the metabolic equivalent of that intensity. The total was obtained in MET minutes/week by summing the intensities. The estimated glomerular filtration rate (eGFR) was calculated using the CKD-Epi equation without correction for race. Our study considered participants to have previously known diabetes at visits 1–3 when answering yes to either “Have you been previously told by a physician that you had/have diabetes (sugar in the blood)?” or “Have you used medication for diabetes in the past two weeks”? 

The ten-year risk of a major cardiovascular event (myocardial infarction, stroke, or cardiovascular death) was estimated for each individual based on age, sex, smoking, systolic blood pressure (SBP), and total cholesterol according to the WHO Risk Chart Working Group table for the Tropical Latin America region [15]. When ≥20%, we categorized it as high. 

We considered targets according to the 2022 American Diabetes Association (ADA) guidelines for therapeutic targets [16,17]. Glucose control was defined when HbA1c was less than 7% (53 mmol/mol) (target A). The targets for adequate blood pressure and LDL-c control varied based on participants’ cardiovascular risk. For those not at high cardiovascular risk, blood pressure (target B) and LDL-c (target C) were <140/90 mmHg and <100 mg/dL (2.58 mmol/L), respectively. Targets of <130/80 mmHg and <70 mg/dL (1.81 mmol/L) were adopted for those already with clinical cardiovascular disease (CVD) or at high risk of developing it [17]. We assessed target achievement based on values obtained at the visit during which diabetes was first ascertained. 

The 2023 ADA guidelines recommended stricter targets for blood pressure (<130/80 mmHg for all) and LDL-c (<70 mg/dL (1.81 mmol/L) and for those with high risk or clinical cardiovascular disease <55 mg/dL (1.42 mmol/L) [18,19]. However, considering the Brazilian context in which most patients with diabetes do not have access to SGLT2 inhibitors, GLP-1 agonists, and the more potent lipid-lowering drugs, this study used the less stringent 2022 ADA goals in primary analyses, performing a sensitivity analysis based on the ADA’s 2023 therapeutic targets. 

### 2.3. Outcomes

**Figure 1:**
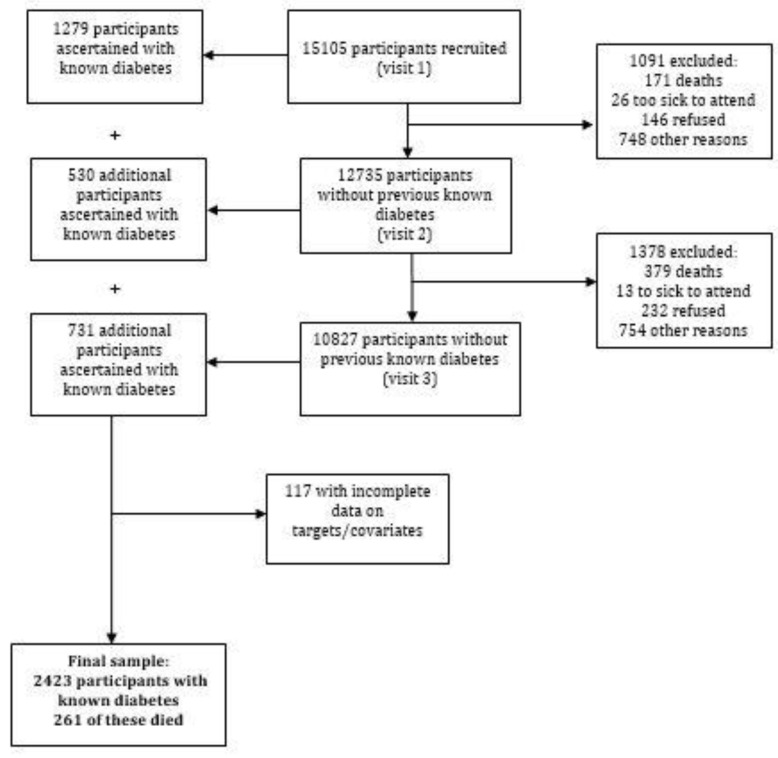
Flow diagram of selected participants with known diabetes. ELSA-Brasil, 2008–2019.

ELSA has conducted annual telephone surveillance since 2009 to ascertain deaths and confirmed them through hospital records and death certificates [20]. Those with known diabetes at Visit 1 were followed from that point onward. Those ascertained with known diabetes at Visits 2 and 3 established their baseline and initiated their follow-up at the visit of diabetes ascertainment (Figure 1). 

Although ELSA-Brasil is still completing adjudication of the cause of ELSA participants’ deaths, the causes of most deaths have been determined. 

### 2.4. Statistical Analyses

Categorical variables were described as frequencies and percentages, and continuous ones as means and standard deviations (SD). Statistical testing of crude associations between target achievement and mortality was performed with the chi-square test when control was assessed categorically and with analysis of variance (ANOVA) when control was assessed using continuous values of the evaluated factors.

Cox proportional hazards models were performed to evaluate the adjusted association between control and all-cause mortality. First, we analyzed the level of control categorically, comparing deaths in participants with values below the target cutoff to those above. Second, to evaluate the risk of death along an equivalent spectrum of degree of control for each of the three treatable prognostic factors, our approach was to transform their continuous values into z-scores. Additionally, to produce a similar z-score reflecting the equivalent level of control of all three factors concomitantly, we summed the HbA1c, SBP, and LDL-cholesterol z-scores and divided this sum by 3. To capture nonlinear relationships between outcome and z-scores, we used restricted cubic splines in the Cox proportional hazards models. For this, three knots were chosen at the 10th, 50th, and 90th quantiles [21]. We additionally investigated the adjusted association of achieving any two or all three targets with specific causes of death, including adjustment for previous CVD and cardiovascular risk when investigating cardiovascular deaths.

All data analyses were performed using the software R (version 1.3. 1056, R Core Team, Vienna, Austria © 2009–2020) [22].

## 3. Results

Of the 15,105 participants at baseline, we ascertained 2540 (16.8%) as having known diabetes during the study visits: 1279 at baseline, 530 at Visit 2, and 731 at Visit 3. After excluding 117 with missing or incomplete data for targets and covariates, 2423 participants remained for analysis (Figure 1). 

As shown in Table 1, among our sample, 1328 (54.8%) were men, 1074 (44.3%) had self-declared white ethnicity, and 1377 (56.8%) had less than a university education. The mean (SD) age was 55.7 (8.6) years. Many (425; 17.5%) were >64 at baseline and thus ≥75 if alive when we closed the follow-up period. Additionally, 1424 (58.8%) reported having private health insurance, 791 (32.6%) related income up to 4 times the Brazilian minimum wage, 2104 (86.8%) were not current smokers, 297 (12.3%) related a history of cardiovascular disease, and an additional 42 (1.73%) had high cardiovascular (CVD) risk. 

Appendix A shows that having no factor under control was more frequent among men, older individuals, blacks, and those with lower educational achievement, without private health insurance, lower income, and with either a history of CVD or high CVD risk.

Table 2 shows that HbA1c was ≤7% (53 mmol/mol) in 1820 (75.1%) participants. SBP was less than 140 mmHg in low-risk or less than 130 mmHg in high-risk individuals in 1795 (74.1%) participants, and LDL-cholesterol less than 100 mg/dL (2.58 mmol/L) in low-risk or less than 70 mg/dL (1.81 mmol/L) in high-risk individuals in 877 (36.2%) participants; both targets A and B were achieved by 1407 (58.1%) participants, but all ABC goals were at or below target in only 507 (20.9%) participants. 

During an average follow-up of 11 (1.8) years, 261 participants died. Of the 147 participants whose cause of death ELSA investigators had adjudicated (Appendix B), 50 had died of cancer and 53 of cardiovascular disease, and the remaining 44 from a wide variety of other diseases and injuries. 

In unadjusted analyses, achieving the glucose target (HR = 0.33; 95%CI 0.26–0.42) and the blood pressure target (HR = 0.51; 95%CI 0.40–0.65) decreased the risk of death. The risk was especially reduced among those with two or more ABC goals reached (HR = 0.26 to 0.32; 95%CI 0.17–0.49). When we adjusted the associations for covariates (Table 3), the risk of death remained lower with HbA1c ≤7% (≤53 mmol/mol) (HR = 0.66; 95%CI 0.50–0.88) and, though not statistically significant, with blood pressure less than 140 mmHg (in low risk) and less than 130 mmHg (in high risk) (HR = 0.78; 0.60–1.02). The same was seen among those who reached any two of the three ABC goals (HR = 0.63; 95%CI 0.42–0.95) and notably so when the two goals were the glycemic and blood pressure ones (HR = 0.54; 95%CI 0.37–0.78). The most significant risks of death were seen when HbA1c reached or exceeded 9% (75 mmol/mol) (HR = 1.97; 95%CI 1.33–2.91) and when SBP was ≥140 mmHg (HR = 1.50; 95%CI 1.04–2.16). 

In contrast, achieving the LDL-c target, which occurred less frequently, was associated with an increased risk of death from all causes in both crude (HR = 1.55; 95%CI 1.21–1.97) and adjusted (HR = 1.44; 95%CI 1.11–1.88) analyses. The risk was especially high among those with LDL-c values <70 mg/dL (1.81 mmol/L) (adjusted HR = 2.19; 95%CI 1.40–3.42). Not smoking also conferred important protection (HR = 0.59; 95%CI 0.42–0.82) in the adjusted analyses.

In subgroup analyses by cause of death (Appendix B), two or more of the three ABC targets numerically decreased the risk of cardiovascular (crude HR = 1.18; 95%CI 0.58–2.42, adjusted HR = 0.82; 95%CI 0.23–2.98) and other cause deaths (crude HR = 0.65; 95%CI 0.29–1.42, adjusted HR = 0.58 95%CI 0.25–1.34), but not cancer deaths (crude: HR= 0.96; 95%CI 0.62–1.49, adjusted HR = 0.98 95%CI 0.63–1.54). However, none of these associations achieved statistical significance. 

**Figure 2:**
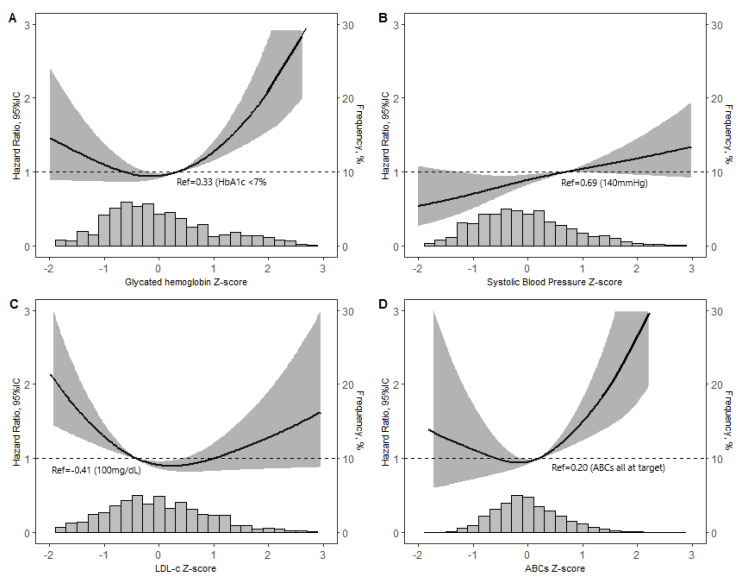
Risk of death (the greyed area is the zone of 95% confidence) according to the z-score of treatable prognostic factors: (**A**) Hba1c, (**B**) systolic blood pressure, (**C**) low-density lipoprotein (LDL)-cholesterol, and (**D**) the overall ABC z-score. Associations were obtained through restricted cubic spline analyses using Cox proportional hazards models adjusted for age, sex, school achievement, race/ethnicity, private health insurance, income, history of cardiovascular disease, smoking, physical activity, duration of diabetes, body mass index, HDL-cholesterol, urine albumin to creatinine ratio, and estimated glomerular filtration rate. The HbA1c z-score was additionally adjusted for LDL-c and systolic blood pressure, the systolic blood pressure z-score additionally for LDL-c and HbA1c, and the LDL-c z-score additionally for HbA1c and systolic blood pressure. The y-axis to the left of each plot indicates the hazard ratio of the risk of death according to the level of the respective modifiable prognostic factor. The histogram at the bottom and the y-axis to the right of each graph display the distribution of z-scores of the variable in question.

Figure 2 presents the risk of death according to the adjusted z-scores of each of the ABCs and of the three ABCs when considered concomitantly, permitting a description of the risk of death across the entire control spectrum. The reference (HR = 1) in these analyses was set at the target value for each factor. Risk increased steeply and linearly above the HbA1c cutoff and was also non-significantly higher at the lowest HbA1c values. Though less in magnitude, the risk increased uniformly across the blood pressure spectrum. Risk for LDL-c was bimodal, reaching a nadir at a z-score equivalent to an LDL-c value of 116 mg/dL (3 mmol/L), being greatest at LDL-c values within target and numerically greater, though not significantly so, at highest LDL-c values. The overall ABC z-score showed slightly greater risk at the low end of the distribution and steeply increased risk at the high end of the composite z-score distribution. 

Considering the new ADA 2023 therapeutic targets for blood pressure and LDL-c, relative protection against all causes of death was slightly (6%) greater than with the 2022 targets when any one or any two ABC goals were reached (HR = 0.67; 95%CI 0.50–0.92 and HR = 0.57; 95%CI 0.40–0.82, respectively). However, those achieving the 2023 LDL-c target showed a further increase in risk (HR = 1.98; 95%CI 1.36–2.90) (Appendix C and Appendix D). 

## 4. Discussion

In this large, free-living sample of middle-aged and elderly Brazilian adults with known diabetes, slightly more than 10% had died over 11 (1.8) years of follow-up. A large fraction reached blood glucose and blood pressure targets, but only slightly more than one-third achieved the LDL-c target. Lower mortality was present when HbA1c was on target, and deaths were halved among those with HbA1c <7% (≤53 mmol/mol) compared to those with HbA1c ≥9% (75 mmol/mol). The risk of death was also reduced when two or more ABC goals had been achieved. However, those reaching the LDL-c target, paradoxically, had greater mortality. 

In addition to the minimal mortality risk seen in well-controlled diabetes in Sweden [2], the Steno-2 Study showed that intensive treatment produced a 46% relative reduction in all-cause and a 57% reduction in cardiovascular deaths [1]. These findings have stimulated intensive control of multiple CVD risk factors. The Swedish results, as ours, indicated that a glycated hemoglobin level outside the target range was the strongest predictor of death [2]. 

Using another approach to estimate benefit, Kianmehr et al. applied risk equations based on the observation of outcomes in the Action to Control Cardiovascular Risk in Diabetes (ACCORD) trial. They observed a considerable benefit in lowering the highest glucose, blood pressure, and LDL-c levels [23]. Compared with an SBP of 160 mmHg, SBP levels of 139 mmHg, 128 mmHg, and 114 mmHg predicted 1.1-, 1.5-, and 1.9-year gains in life expectancy, respectively. Similarly, LDL-c levels of 107 mg/dL (2.76 mmol/L), 84 mg/dL (2.17 mmol/L), and 59 mg/dL (1.52 mmol/L) predicted 0.5-, 0.7-, and 0.9-year gains when compared with an LDL-c of 146 mg/dL (3.77 mmol/L). An HbA1c of 7.7% (61 mmol/mol) predicted a 3.4-year gain when compared to a level of 9.9% (85 mmol/mol), with little additional benefit at lower levels [23]. 

Of note, however, the benefits of the ABC control in most other studies are much less evident. Meta-analysis of trials showed only an 18% relative reduction in all-cause mortality and a 28% relative reduction in cardiovascular deaths [24]. Additionally, the benefit is less certain in elderly patients. A Japanese trial in 1173 patients with a mean age of 72, aiming for HbA1c <6.9% (52 mmol/mol), SBP <130 mmHg, and LDL-c <100 mg/dL (2.58 mmol/L), among other targets, found no benefit of intensifying treatment [25]. 

Despite the discrepancy in the assessed benefits, authorities in Europe and the United States (e.g., the ADA) have recommended increasingly intensive treatment. In so doing, they indicate the need to personalize glycemic control and, more recently, to select the classes of anti-diabetic medication prescribed depending on individual patient risks and necessities [18]. The European Society of Cardiology (ESC) and the European Association for the Study of Diabetes (EASD) recommend HbA1c <7% (53 mmol/mol) with individualized exceptions, blood pressure <130 mmHg, and an LDL-c <100 mg/dL (2.58 mmol/L), <70 md/dL (1.81 mmol/L) and <55 mg/dL (1.42 mmol/L) for moderate, high and very high CVD risk [26]. The UK National Institute for Health and Care Excellence (NICE) recommends HbA1c <7% (53 mmol/mol) with individualized exceptions [27], blood pressure <135/85 mmHg for adults under 80 and <145/85 mmHg for adults 80 and over [28], and atorvastatin 20 mg for those who have a 10% or greater 10-year risk of developing CVD [29].

Extrapolating from trials to the real world is not simple. Trial participants frequently differ considerably from those with diabetes in free-living populations. Additionally, procedures and health care personnel in trials generally differ from those of usual care. Thus, real-world data are essential to validate trial results in the settings where they are applied. In this regard, it is reassuring that our findings support recommendations to achieve blood pressure and especially glucose targets. Mortality in diabetes has shifted over recent decades from being principally cardiovascular to being due to a broader category of causes [30]. Thus, it makes sense that control of glycemia, a risk factor for both cardiovascular and non-cardiovascular deaths, showed a more predominant role in preventing overall deaths than hypertension and LDL-c, as these factors, especially the latter, are more related to cardiovascular outcomes. 

It is important to note that our LDC-c results are in discordance with the perceived benefit of the clinical trial literature. Among those with diabetes and known cardiovascular disease, these trials have documented significant clinical gain from treating hyperlipidemia, including reducing all-cause mortality. Meta-analyses of trials in low-risk individuals with diabetes, however, have produced conflicting results, the most recent showing a non-statistically significant advantage for those treated with statins in all-cause mortality [31,32,33,34]. A recent study of primary prevention of cardiovascular disease through lipid lowering with bempedoic acid, in which approximately two-thirds of participants had diabetes, did show significant risk reduction (HR = 0.70; 95%CI 0.55–0.89) [34]. In contrast, many observational studies of low-risk patients with diabetes, like ours, report findings of greater risk for all-cause mortality for those with LDL-c in the target range. The Translating Research Into Action for Diabetes (TRIAD) Study was a multicenter, prospective, observational study of 8733 patients with diabetes treated in managed care in the U.S. Among its participants, being within an LDL-c target rather than having dyslipidemia was associated with higher all-cause and cardiovascular mortality [35]. Chiang et al. [36] found a nadir of mortality in outpatients with diabetes at values between 100 and 130 mg/dL of LDL-c (2.58 mmol/L and 3.36 mmol/L), with increased mortality below 70 mg/dL (1.81 mmol/L). A National Health and Nutrition Examination Survey (NHANES) follow-up study [37] with a sample representative of the U.S. population also found greater all-cause mortality risk at the lowest LDL-c levels. 

Thus, our findings highlight the need for additional studies to understand the association of achieving LDL-c targets with greater mortality among those with diabetes but not characterized as having higher cardiovascular risk. Hopefully, they can clarify whether low LDL-c values in patients with diabetes but without higher CVD risk represent a real risk or derive from unexplained confounding or reverse causality, i.e., with lower LDL-c values resulting from underlying disease at baseline rather than causing the increase in deaths observed [38].

Our finding that 20.9% of subjects achieved all of the three ABC goals obtained from a cohort of employees of universities and research institutions cannot evaluate the fraction of Brazilian adults with diabetes achieving control targets. However, Reis et al. [6], analyzing nationally representative Brazilian data using 2021 ADA targets, showed that only 46% of adults with diabetes achieved the HbA1c target, 51.4% the blood pressure target, 40% the LDL-c target, and only 12.5% all three targets. Findings, mostly from high-income countries, though often documenting somewhat better target achievement, also show significant gaps between the obtained and desired levels of control [39,40,41]. Based on our findings and the literature in general, at least for the glycemic and blood pressure goals, major efforts should be made to increase the currently low fraction of those with diabetes at target in the population.

While focusing on the ABC goals, we recognize the importance of other treatments. With respect to drug therapy, emphasis is increasingly being placed on the use of more recent anti-diabetic medications of greater proven benefit. Nutritional medical therapy can produce major benefits in terms of control of weight loss/normalization, glycemia, dyslipidemia, and blood pressure. It should, along with increased physical activity, be an integral part of diabetes care [42,43,44]. Also, the growing availability of data collected by health systems to support clinical practice can facilitate efforts to improve control. Providing such feedback on control in a structured way was associated with a 66% reduction in mortality in patients with diabetes in Hong Kong [45]. With increasing access to electronic health records, feedback of such data is increasingly possible. A diabetes clinical decision support system based on such feedback has been shown to improve glucose and blood pressure control and reduce cardiovascular risk in the United States. This system, as reported in 2019, was already in use in health systems, covering more than 3 million patients [46]. 

The potential limitations of our study merit note. Our sample comprises active or retired civil servants who are more socially privileged than the general Brazilian population. However, our sample included a broad spectrum of the principal relevant domains of the Brazilian population with diabetes (age, sex, ethnicity, educational achievement, income, adiposity, CVD risk, private health insurance coverage). Moreover, representativeness is generally not a major issue in studying longitudinal associations such as those reported here [47]. The lack of data in ELSA-Brasil for retinopathy and other diabetes-related complications limits our ability to adjust for these covariates. However, given our adjustments for cardiovascular and renal disease, additional adjustments for retinopathy and other complications would have likely resulted in little change in the hazard ratios. Finally, the small number of deaths and thus limited statistical power to analyze relative mortality within the specific cause groups requires that findings for these groups be viewed as preliminary.

The strengths of our study include its contemporary, free-living sample of participants residing in multiple locations across Brazil, its careful and extensive collection of data on the targets and their covariates, and its standardized and centralized laboratory measurements. 

## 5. Conclusions

In conclusion, control of the modifiable risk factors hyperglycemia and hypertension produces clinically relevant decreases in all-cause mortality. Given this finding, there is much room for improvement in controlling these risk factors in diabetes in Brazil and worldwide. The conflicting evidence regarding the benefit demonstrated in lipid-lowering trials and the greater risk with low LDL-c in many observational studies indicates the need for further investigation of both the possible reasons for the observational findings and the real benefit of pharmacologically lowering lipids in contemporary individuals with diabetes at lower CVD risk.

## Figures and Tables

**Table 1:** Baseline socio-demographic and clinical characteristics of 2423 adults with known diabetes. ELSA-Brasil. 2008 to 2019.

	All	Alive	Died	*p* *
Chacteristic	N = 2423 n (%)	N = 2162(89.2%) n (%)	N = 261(10.7%) n (%)	
Sex				<0.001
Male	1328 (54.8)	1139 (85.8)	189 (14.2)	
Female	1095 (45.2)	1023 (93.4)	72 (6.58)	
Age (years)				<0.001
44 to 54	1112 (45.9)	1060 (95.3)	52 (4.68)	
55 to 64	886 (36.6)	768 (86.7)	118 (13.3)	
>64	425 (17.5)	334 (78.6)	91 (21.4)	
Ethnicity				0.344
Black	505 (20.8)	439 (86.9)	66 (13.1)	
*Pardo*	688 (28.4)	618 (89.8)	70 (10.2)	
White	1074 (44.3)	968 (90.1)	106 (9.87)	
Asian	127 (5.24)	111 (87.4)	16 (12.6)	
Indigenous	29 (1.20)	26 (89.7)	3 (10.3)	
Education				<0.001
Less than university	1377 (56.8)	1176 (85.4)	201 (14.6)	
University	1046 (43.2)	986 (94.3)	60 (5.74)	
Private health insurance				<0.001
Yes	1424 (58.8)	1315 (92.3)	109 (7.65)	
No	999 (41.2)	847 (84.8)	152 (15.2)	
Income (minimum wages) **				<0.001
<4	884 (36.5)	786 (88.9)	98 (11.1)	
4 to 7	791 (32.6)	682 (86.2)	109 (13.8)	
≥8	748 (30.9)	694 (92.8)	54 (7.22)	
BMI (kg/m^2^)				0.061
<25	408 (16.8)	351 (86.0)	57 (14.0)	
25–29.9	994 (41.0)	885 (89.0)	109 (11.0)	
30–34.9	689 (28.4)	621 (90.1)	68 (9.87)	
≥35	332 (13.7)	305 (91.9)	27 (8.13)	
Current smoking				0.019
Yes	319 (13.2)	272 (85.3)	47 (14.7)	
No	2104 (86.8)	1890 (89.8)	214 (10.2)	
Physical activity (MET min/wk) M (SD)	559 (942)	558 (940)	568 (963)	0.875
Waist hip ratio M (SD)	0.97 (0.08)	0.97 (0.08)	0.98 (0.07)	0.004
Albumin:creatinine ratio (mg/g) M (SD)	43.4 (249)	23.5 (106)	208 (672)	<0.001
eGFR (mL/min per 1.73 m^2^ M (SD)	81.7 (16.5)	82.7 (15.7)	73.4 (20.0)	<0.001
History of CVD				<0.001
Yes	297 (12.3)	244 (82.2)	53 (17.8)	
No	2126 (87.7)	1918 (90.2)	208 (9.78)	
WHO CVD risk ***				<0.001
High risk	28 (1.32)	17 (60.7)	11 (39.3)	
Low risk	2098 (98.7)	1901 (90.6)	197 (9.39)	
GLP-1RA or SGLT2i use at baseline				<0.001
Yes	25 (1.03)	19 (0.78)	6 (0.24)	
No	2398 (98.9)	2140 (89.2)	258 (10.7)	

n (%) unless otherwise indicated; M (SD) = Mean (Standard Deviation); BMI = body mass index; MET = metabolic equivalent of task; eGFR = Estimated glomerular filtration rate; CVD = cardiovascular disease; WHO = World Health Organization/GLP-1RA = GLP-1 receptor agonist; SGLT2i = sodium glucose cotransport 2 inhibitor. * Chi-square test for statistical significance of categorical variables and ANOVA for continuous ones of the difference in variable level or frequency. ** The monthly minimum wage was BRL 986.00 at the study midpoint. Numbers are multiples of this wage. *** Only among participants without a history of CVD.

**Table 2:** Clinical characteristics and prognostic factors at the beginning of follow-up with death among 2423 adults with diabetes. ELSA-Brasil 2008 to 2019.

	All	Alive	Died	*p* *
Targets and Statin Use	N = 2423 n (%)	N = 2162(89.2%) n (%)	N = 261(10.7%) n (%)	
Glucose target achieved				<0.001
Yes (HbA1c < 7%)	1820 (75.1)	1683 (92.5)	137 (7.53)	
No	603 (24.9)	479 (79.4)	124 (20.6)	
Blood pressure target achieved				<0.001
Yes (on target)	1795 (74.1)	1636 (91.1)	159 (8.86)	
No	628 (25.9)	526 (83.8)	102 (16.2)	
LDL-c target achieved				0.005
Yes (on target)	877 (36.2)	757 (86.3)	120 (13.7)	
No	1546 (63.8)	1405 (90.9)	141 (9.12)	
AB targets achieved				<0.001
0	215 (8.87)	161 (74.9)	54 (25.1)	
1	801 (33.1)	683 (85.3)	118 (14.7)	
2	1407 (58.1)	1318 (93.7)	89 (6.33)	
ABC targets achieved				<0.001
0	143 (5.90)	107 (74.8)	36 (25.2)	
1	575 (23.7)	492 (85.6)	83 (14.4)	
2	1198 (49.4)	1105 (92.2)	93 (7.76)	
3	507 (20.9)	458 (90.3)	49 (9.66)	
HbA1c (%)				<0.001
<6	1179 (48.7)	1094 (92.8)	85 (7.21)	
6 to 6.4	409 (16.9)	384 (93.9)	25 (6.11)	
6.5 to 6.9	232 (9.57)	205 (88.4)	27 (11.6)	
7 to 7.9	256 (10.6)	211 (82.4)	45 (17.6)	
8 to 8.9	131 (5.41)	107 (81.7)	24 (18.3)	
≥9	216 (8.91)	161 (74.5)	55 (25.5)	
Systolic blood pressure (mmHg)				<0.001
<120	870 (35.9)	809 (93.0)	61 (7.01)	
120 to 129	605 (25.0)	554 (91.6)	51 (8.43)	
130 to 139	443 (18.3)	390 (88.0)	53 (12.0)	
140 to 159	390 (16.1)	322 (82.6)	68 (17.4)	
≥160	115 (4.75)	87 (75.7)	28 (24.3)	
LDL-cholesterol (mg/dL)				<0.001
<55	67 (2.77)	52 (77.6)	15 (22.4)	
55 to 69	175 (7.22)	141 (80.6)	34 (19.4)	
70 to 99	642 (26.5)	568 (88.5)	74 (11.5)	
100 to 129	739 (30.5)	684 (92.6)	55 (7.44)	
130 to 159	517 (21.3)	465 (89.9)	52 (10.1)	
≥160	283 (11.7)	252 (89.0)	31 (11.0)	
Statin use				0.982
Yes	950 (39.2)	847 (89.2)	103 (10.8)	
No	1473 (60.8)	1315 (89.3)	158 (10.7)	

n (%) unless otherwise indicated; LDL-c = low-density lipoprotein cholesterol; HbA1c = glycated hemoglobin; ABC = HbA1c, blood pressure and LDL-c. AB = HbA1c and blood pressure. * Chi-square test for statistical significance of the difference in variable level.

**Table 3:** Crude and adjusted * risk of death according to the level of treatable prognostic factors among individuals with known diabetes. ELSA-Brasil 2008 to 2019.

	Crude	Adjusted *	
Characteristic	HR 95%CI	HR 95%CI	*p*
Glucose target reached	0.33 (0.26–0.42)	0.66 (0.50–0.88)	**0.004**
Blood pressure target reached	0.51 (0.40–0.65)	0.78 (0.60–1.02)	0.069
LDL-c target reached	1.55 (1.21–1.97)	1.44 (1.11–1.88)	**0.006**
Non-smoking	0.67 (0.49–0.92)	0.59 (0.42–0.82)	**0.002**
ABC targets reached (reference: 0)			
1	0.49 (0.33–0.73)	0.81 (0.54–1.22)	0.323
2 **	0.26 (0.17–0.38)	0.63 (0.42–0.95)	**0.027**
3	0.32 (0.21–0.49)	0.75 (0.47–1.19)	0.222
AB targets reached (reference: 0)			
1	0.52 (0.38–0.72)	0.79 (0.57–1.11)	0.179
2	0.21 (0.15–0.29)	0.54 (0.37–0.78)	**0.001**
HbA1c (%; reference: ≤6)			
6 to 6.4	0.87 (0.56–1.36)	0.64 (0.40–1.00)	0.051
6.5 to 6.9	1.72 (1.12–2.65)	0.95 (0.60–1.51)	0.831
7 to 7.9	2.76 (1.92–3.96)	1.19 (0.80–1.78)	0.388
8 to 8.9	2.80 (1.78–4.41)	0.91 (0.56–1.48)	0.701
≥9	4.14 (2.95–5.82)	1.97 (1.33–2.91)	**0.001**
SBP (mmHg; reference: ≤120)			
120 to 129	1.22 (0.84–1.77)	0.90 (0.61–1.32)	0.575
130 to 139	1.77 (1.23–2.56)	1.32 (0.90–1.92)	0.156
140 to 159	2.73 (1.93–3.85)	1.50 (1.04–2.16)	**0.029**
≥160	4.15 (2.65–6.49)	1.15 (0.71–1.86)	0.578
LDL-c (mg/dL; reference: 100 to 130)			
<55	3.33 (1.88–5.89)	2.48 (1.38–4.47)	**0.003**
55 to 69	2.83 (1.85–4.34)	2.19 (1.40–3.42)	**0.001**
70 to 99	1.59 (1.12–2.26)	1.42 (1.00–2.03)	0.051
130 to 159	1.38 (0.95–2.02)	1.23 (0.83–1.82)	0.298
≥160	1.49 (0.96–2.31)	1.53 (0.97–2.41)	0.068
Statin use	0.99 (0.77–1.27)	0.99 (0.75–1.29)	0.920
In Low CVD Risk	0.98 (0.76–1.27)	0.99 (0.75–1.30)	0.933
In High CVD Risk	1.08 (0.41–2.82)	1.12 (0.41–3.06)	0.828

LDL-c = low-density lipoprotein cholesterol; HbA1c = glycated hemoglobin; SBP = systolic blood pressure; ABC = HbA1c, blood pressure, and LDL-c; AB = HbA1c and blood pressure; CVD = cardiovascular disease. * through Cox proportional hazards regression adjusting for age, sex, educational achievement, ethnicity, private health insurance, income, smoking, physical activity, HDL-cholesterol, body mass index, diabetes incidence, history of cardiovascular disease, urine albumin to creatinine ratio, estimated glomerular filtration rate, and additionally for HbA1c, LDL-c, and systolic blood pressure (SBP), except when one of these is the exposure being analyzed. ** Any two of the three targets.

## Data Availability

Due to considerations noted in our approval by the local ethical committees, the data used in this study can be made available for research purposes by request to ELSA’s Datacenter (estatisticaelsa@gmail.com) and the ELSA Publications Committee. ELSA-Brasil was registered at ClinicalTrials.gov as NCT02320461.

## Appendix A

**Table A1:** Baseline socio-demographic and clinical characteristics of 2423 adults with known diabetes according to ABC targets achieved. ELSA-Brasil. 2008 to 2019.

ABC Targets Achieved						
	All	0 Targets	1 Target	2 Targets	3 Targets	*p* *
Characteristic	N = 2423 n (%)	N = 143 (5.90%) n (%)	N = 575 (23.7%) n (%)	N = 1198 (49.4%) n (%)	N = 507 (20.9%) n (%)	
Sex						<0.001
Male	1328 (54.8)	96 (7.23)	353 (26.6)	592 (44.6)	287 (21.6)	
Female	1095 (45.2)	47 (4.29)	222 (20.3)	606 (55.3)	220 (20.1)	
Age (years)						<0.001
44 to 54	1112 (45.9)	43 (3.87)	257 (23.1)	596 (53.6)	216 (19.4)	
55 to 64	886 (36.6)	62 (7.00)	219 (24.7)	424 (47.9)	181 (20.4)	
>64	425 (17.5)	38 (8.94)	99 (23.3)	178 (41.9)	110 (25.9)	
Ethnicity						<0.001
Black	505 (20.8)	53 (10.5)	137 (27.1)	240 (47.5)	75 (14.9)	
White	688 (28.4)	58 (8.43)	179 (26.0)	329 (47.8)	122 (17.7)	
*Pardo*	1074 (44.3)	26 (2.42)	218 (20.3)	563 (52.4)	267 (24.9)	
Asian	127 (5.24)	6 (4.72)	31 (24.4)	52 (40.9)	38 (29.9)	
Indigenous	29 (1.20)	0 (0.00)	10 (34.5)	14 (48.3)	5 (17.2)	
Education						<0.001
Less than university	1377 (56.8)	122 (8.86)	380 (27.6)	642 (46.6)	233 (16.9)	
University	1046 (43.2)	21 (2.01)	195 (18.6)	556 (53.2)	274 (26.2)	
Private health insurance						<0.001
Yes	1424 (58.8)	58 (4.07)	294 (20.6)	731 (51.3)	341 (23.9)	
No	999 (41.2)	85 (8.51)	281 (28.1)	467 (46.7)	166 (16.6)	
Income (minimum wages) **						<0.001
≤4	884 (36.5)	61 (6.90)	251 (28.4)	409 (46.3)	163 (18.4)	
4 to 7	791 (32.6)	66 (8.34)	198 (25.0)	400 (50.6)	127 (16.1)	
≥8	748 (30.9)	16 (2.14)	126 (16.8)	389 (52.0)	217 (29.0)	
Current smoking						0.016
Yes	319 (13.2)	15 (4.70)	89 (27.9)	167 (52.4)	48 (15.0)	
No	2104 (86.8)	128 (6.08)	486 (23.1)	1031 (49.0)	459 (21.8)	
BMI (kg/m^2^)						0.766
<25	408 (16.8)	19 (4.66)	86 (21.1)	215 (52.7)	88 (21.6)	
25–29.9	994 (41.0)	61 (6.14)	232 (23.3)	491 (49.4)	210 (21.1)	
30–34.9	689 (28.4)	41 (5.95)	166 (24.1)	334 (48.5)	148 (21.5)	
35≥40	332 (13.7)	22 (6.63)	91 (27.4)	158 (47.6)	61 (18.4)	
Waist hip ratio M (SD)	0.97 (0.08)	0.99 (0.08)	0.98 (0.07)	0.96 (0.08)	0.97 (0.08)	<0.001
Albumin:creatinine ratio (mg/g) M (SD)	43.4 (249)	200 (561)	60.1 (328)	23.6 (147)	26.8 (159)	<0.001
eGFR (mL/min per 1.73 m^2^ M (SD)	81.7 (16.5)	78.2 (16.9)	81.3 (16.8)	82.7 (15.9)	80.9 (17.2)	<0.001
WHO CVD risk ***						<0.001
High risk	28 (1.32)	15 (53.6)	11 (39.3)	2 (7.14)	0 (0.00)	
Low risk	2098 (87.7)	105 (5.00)	492 (23.5)	1052 (50.2)	446 (21.3)	
History of CVD						<0.001
Yes	297 (12.3)	26 (8.75)	79 (26.6)	111 (37.4)	81 (27.3)	
No	2126 (87.7)	117 (5.50)	498 (23.3)	1087 (51.1)	426 (20.0)	

n (%) unless otherwise indicated; M (SD) = Mean (Standard Deviation); ABC = HbA1c, blood pressure and LDL-c; BMI = body mass index; CVD = cardiovascular disease; eGFR = Estimated glomerular filtration rate; WHO = World Health Organization. Row percentages are presented for the number of targets achieved columns. * Chi-square test for statistical significance of categorical variables and ANOVA for continuous ones of the difference in variable level or frequency. ** The monthly minimum wage was BRL 986.00 at the time of the study. Numbers are multiples of this wage. *** Only among participants without a history of CVD.

## Appendix B

**Table A2:** Distribution of causes of death and crude and adjusted * risk of death for those achieving any two or all three ABC targets among 147 individuals with known diabetes and adjudicated outcomes. ELSA-Brasil, 2008 to 2019.

		≥2 ABC Targets Achieved		
		Crude	Adjusted *	
Cause of Death	N (%)	HR 95%CI	HR 95%CI	*p*
Cardiovascular diseases	53 (36.1)	1.18 (0.58–2.42)	0.82 (0.23–2.98)	0.591
Cancer	50 (34.0)	0.96 (0.62–1.49)	0.98 (0.63–1.54)	0.662
Other	44 (29.9)	0.65 (0.29–1.42)	0.58 (0.25–1.34)	0.202
Digestive diseases	11 (7.4)			
Chronic kidney diseases	8 (5.4)			
Injuries	8 (5.4)			
Chronic respiratory diseases	4 (2.7)			
Miscellaneous **	13 (8.8)			

ABC = HbA1c, blood pressure and LDL-c. * through Cox proportional hazards regression adjusting for age, sex, smoking, and in the cardiovascular disease deaths analysis, additionally for a history of cardiovascular disease and for CVD risk as determined by the appropriate WHO chart ** blood, neurological, mental (suicide), and musculoskeletal diseases.

## Appendix C

**Table A3:** Unadjusted associations of prognostic factors at the beginning of follow-up with death among 2423 adults with diabetes according to ADA 2023 goals. ELSA-Brasil, 2008 to 2019.

	All	Alive	Died	*p* *
Target Achieved	N = 2423 n (%)	N = 2162 (89.2%) n (%)	N = 261 (10.7%) n (%)	
Blood pressure				<0.001
Yes	1132(46.7)	1043 (92.1)	89 (7.86)	
No	1291 (53.3)	1119 (86.7)	172 (13.3)	
LDL-c				0.005
Yes	246 (9.87)	191 (79.9)	48 (20.1)	
No	2184 (90.1)	1971 (90.2)	213 (9.75)	
Number of ABC targets achieved				<0.001
0	347 (14.3)	271 (78.1)	76 (21.9)	
1	1050 (43.3)	940 (89.5)	110 (10.5)	
2	937 (38.7)	876 (93.5)	61 (6.51)	
3	89 (3.67)	75 (84.3)	14 (15.7)	

LDL-c = low-density lipoprotein cholesterol; ABC = HbA1c, blood pressure and LDL-c. * Chi-square test for statistical significance of the difference in variable level.

## Appendix D

**Table A4:** Crude and adjusted * risk of death from all causes according to the level of treatable prognostic factors among individuals with known diabetes. ELSA-Brasil between 2008 and 2019, according to ADA 2023 treatment goals.

	Death		
	Crude	Adjusted *	
Target Achieved	HR 95%CI	HR 95%CI	*p*
Blood pressure	0.57 (0.44–0.73)	0.72 (0.56–0.93)	**0.013**
LDL-c	2.21 (1.62–3.02)	1.98 (1.36–2.90)	**<0.001**
Number of ABC targets (reference: 0)			
1	0.49 (0.33–0.73)	0.67 (0.50–0.92)	**0.013**
2 **	0.26 (0.17–0.38)	0.57 (0.40–0.82)	**0.002**
3	0.32 (0.21–0.49)	0.93 (0.52–1.67)	0.806

ABC = HbA1c, blood pressure and LDL-c. LDL-c = low-density cholesterol. * through Cox proportional hazards regression adjusting for age, sex, educational achievement, ethnicity, private health insurance, income, smoking, physical activity, HDL-cholesterol, body mass index, diabetes incidence, history of cardiovascular disease, urine albumin to creatinine ratio, estimated glomerular filtration rate and additionally for HbA1c, LDL-c, and systolic blood pressure, except when one of these is the exposure being analyzed. ** Any two of the three targets.
